# Multichannel Convolutional Neural Network for Biological Relation Extraction

**DOI:** 10.1155/2016/1850404

**Published:** 2016-12-07

**Authors:** Chanqin Quan, Lei Hua, Xiao Sun, Wenjun Bai

**Affiliations:** ^1^Graduate School of System Informatics, Kobe University, Kobe, Japan; ^2^Department of Computer and Information Science, Hefei University of Technology, Hefei, China

## Abstract

The plethora of biomedical relations which are embedded in medical logs (records) demands researchers' attention. Previous theoretical and practical focuses were restricted on traditional machine learning techniques. However, these methods are susceptible to the issues of* “vocabulary gap”* and data sparseness and the unattainable automation process in feature extraction. To address aforementioned issues, in this work, we propose a multichannel convolutional neural network (MCCNN) for automated biomedical relation extraction. The proposed model has the following two contributions: (1) it enables the fusion of multiple (e.g., five) versions in word embeddings; (2) the need for manual feature engineering can be obviated by automated feature learning with convolutional neural network (CNN). We evaluated our model on two biomedical relation extraction tasks: drug-drug interaction (DDI) extraction and protein-protein interaction (PPI) extraction. For DDI task, our system achieved an overall *f*-score of 70.2% compared to the standard linear SVM based system (e.g., 67.0%) on DDIExtraction 2013 challenge dataset. And for PPI task, we evaluated our system on Aimed and BioInfer PPI corpus; our system exceeded the state-of-art ensemble SVM system by 2.7% and 5.6% on *f*-scores.

## 1. Introduction

DDI and PPI are two of the most typical tasks in the field of biological relation extraction. DDI task aims to extract the interactions among two or more drugs when these drugs are combined and act with each other in human body; the hidden drug interactions may seriously affect the health of human body. Therefore, it is significant to further understand the interactions of drugs to reduce drug-safety accidents. Different from DDI task, PPI task aims to extract the interaction relations among proteins, and it has captured much interest among the study of biomedical relations recently [1, 2]. There are a number of databases which have been created for DDI (DrugBank [3, 4]) and PPI (MINT [5], IntAct [6]). However, with the rapid growth of biomedical literatures (e.g., MedLine has doubled in size within decade), it is hard for these databases to keep up with the latest DDI or PPI. Consequently, efficient DDI and PPI extraction systems become particularly important.

Previous studies have explored many different methods for DDI and PPI tasks. The dominant techniques generally fall under three broad categories: cooccurrence based method [7], rule-pattern based method [8, 9], and statistical machine learning (ML) based method [10–13]. Cooccurrence based method considers two entities interacting with each other if entities occur in the same sentence. A major weakness of this method is its tendency for having a high recall but a low precision.

The rule and pattern based methods employ predefined patterns and rules to match the labeled sequence. Although having achieved high accuracy among traditional rule and pattern based methods, their sophistication in pattern design and attenuated recall performance deviate them from practical usage. Besides the rule and pattern based methods, ML based techniques view DDI or PPI task as a standard supervised classification problem, that is, to decide whether there is an interaction (binary classification) or what kinds of relations (multilabel classification) between two entities. Compared with cooccurrence and rule-pattern based methods, ML based methods show much better performance and generalization, and the state-of-the-art results for DDI [14] and PPI [2] are all achieved by ML based methods.

Traditional ML based methods usually collect words around target entities as key features, such as unigram, bigram, and trigram, and then these features are put into a* bag-of-words* model and encoded into* one-hot* (https://en.wikipedia.org/wiki/One-hot) type representations; after that, these representations are fed to a traditional classifier such as SVM. However, such representations are unable to capture semantic relations among words or phrases and fail in generalizing the long context dependency [15]. The former issue is rendered as* “vocabulary gap”* (e.g., the words* “depend”* and* “rely”* (these words are considered as the cue words or interaction verbs [8] which are important in biomedical relation extraction) are different in* one-hot* representations, albeit their similar linguistic functions). The latter one is introduced due to the *n*-order Markov restriction that attempts to alleviate the issue of* “curse of dimensionality*.*”* Moreover, the inability to extract features automatically leads to the laborious manual efforts in designing features, which hinders the practical use of traditional ML based methods in extracting biomedical relation features.

To tackle these issues, in this work, we employ word embedding [16, 17] (also known as distribution representations) to represent the words. Different from* one-hot* representation, word embedding could map words to dense vectors of real numbers in a low-dimensional space, and thus the* “vocabulary gap”* problem can be well solved by the dot product of two word vectors. Compared to* one-hot* model, which merely allows the binary coding fashion in words (e.g., yes or no), our employment of the word embedding was able to output the similarity of two words via dot product. Such representation also yield neurological underpinning and is more in consistent with the way of human thinking.

Based on the previous researches on word embedding, this research builds a model on distributed word embedding and proposes a multichannel convolutional neural network (MCCNN) for biomedical relation extraction. The concept* “channel”* in MCCNN is inspired by three-channel RGB image processing [18], which means different word embedding represents different channel and different aspect of input words. The proposed MCCNN integrates different versions of word embeddings for better representing the input words. The only input for MCCNN is the sentences which contain drug-drug pairs (in DDI task) and protein-protein pairs (in PPI task). By looking up different versions of word embedding, input sentences will be initialized and transformed into multichannel representations. After that, the robust neural network method (CNN) will be applied to automatically extract features and feed them to a Softmax layer for the classification.

In sum, our proposed MCCNN model has yield threefold contributions:

(1) We propose a new model MCCNN to tackle DDI and PPI tasks and demonstrate that MCCNN model which relies on multichannel word embedding is effective in extracting biomedical relations features; the proposed model allows the automated feature extraction process. We tested our proposed model on DDIExtraction 2013 challenge dataset and achieved an overall *f*-score 70.2% that outperformed the current best system in DDIExtraction challenge by 5.1% and recent [14] state-of-the-art linear SVM based method by 3.2%.
(2) We also evaluated the proposed model on Aimed and BioInfer PPI extraction tasks. The attained *F*-scores 72.4% and 79.6% which outperform the state-of-the-art ensemble SVM system by 2.7% and 5.6%, respectively.
(3) We release our code (https://github.com/coddinglxf/DDI) taking into account the model's simplicity and good performance.

In remaining sections, Section 2 details proposed MCCNN methods, Section 3 demonstrates and discusses the experiments results, Section 4 briefly concludes this work, and Section 5 details the implementation of MCCNN.

## 2. Method

In this section, firstly, we briefly describe the concept and training algorithm for word embedding. And then, we introduce the multichannel word embedding and CNN model for relation extraction in detail; at last, we show how to train proposed MCCNN model.

### 2.1. Word Embedding

**Figure 1:**
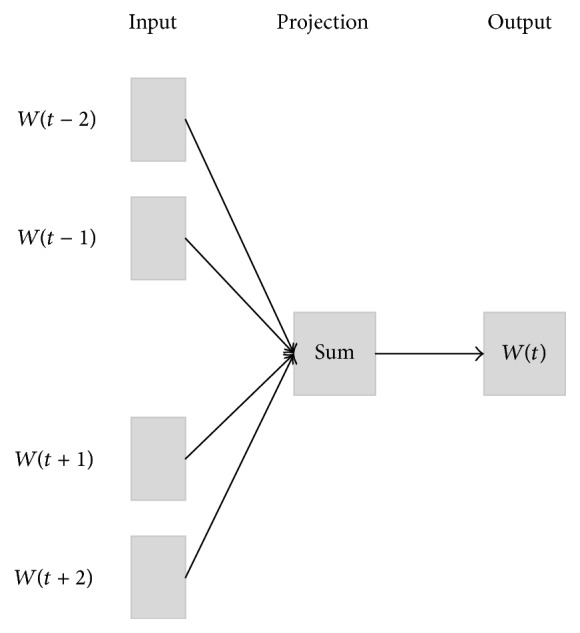
The architecture of CBOW model [17].

Word embedding which could capture both syntactical and semantic information from a large unlabeled corpus has shown its effectiveness in many NLP tasks. The basic assumption for word embedding is that words which occur in similar contexts tend to have similar meanings. Many models had been proposed to train the word embedding, such as NNLM [16], LBL [19], Glove [20], and CBOW. CBOW model (also known as a part of word2vec [17] (https://code.google.com/archive/p/word2vec/)) is employed to train our own word embedding in this work due to its simplicity and effectiveness. CBOW model takes the average embedding of the context words as the context representation, and it reduces the training time by replacing the last traditional Softmax layer with a hierarchical Softmax. In addition, CBOW could further reduce time consumption by negative samples. An outline architecture of CBOW is shown by Figure 1.

### 2.2. Multichannel Word Embedding Input Layer

Word embedding reflects the distributions of words in unlabeled corpus. In order to ensure the maximum coverage of the word embeddings, the articles from PubMed, PMC, MedLine, and Wikipedia are used for training word embedding. Five versions of word embedding are generated based on these corpora. The first four word embeddings are released by Pyysalo et al. [21], while the fifth word embedding is trained by CBOW on MedLine corpus (http://www.nlm.nih.gov/databases/journal.html) (see Figure 1 for more details). The statistics of the five word embeddings are rendered in Table 1.

There are several advantages to use multichannels word embeddings. (1) PMC, MedLine, and PubMed corpus cover most of the literatures in the field of biology; thus these word embeddings can in large extent be used to extract biomedical relation features. (2) Some frequent words may occur in all of the five word embeddings, such kind of words has more information (weight) to leverage. (3) Word information can be shared among different word embeddings. Multichannel word embeddings could enlarge the coverage of vocabulary based on different ways of word embedding and decrease the number of unknown words.

**Figure 2:**
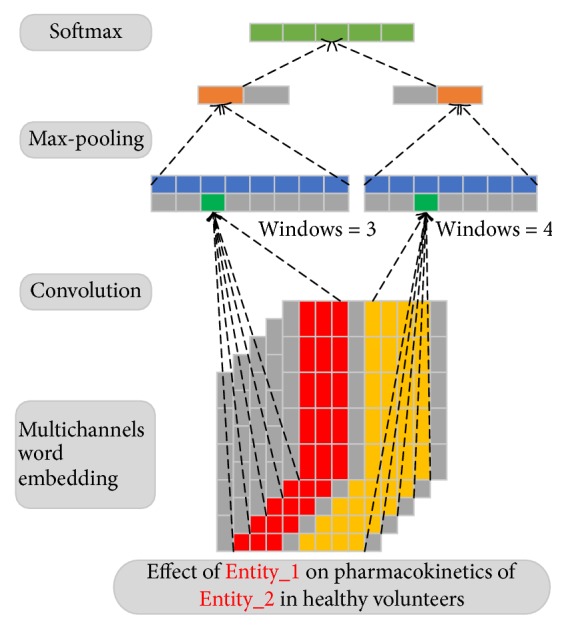
The architecture of the proposed MCCNN. In this example, the length of input sentence is 10, the input word embedding dimension is 5, and there are 5-word embedding channels. Therefore, the size of multichannel inputs is 5 × 10 × 5. Two windows sizes 3 and 4 are used in this example. The green part is generate by (1). The orange part, representing the max-pooling result, is generated by take the maximum value of the blue part through (3). Since there are 2 filters for each window size, 2 features are produced. These extracted features are then concatenated together and fed to a Softmax layer for classification.

The architecture of our proposed MCCNN is showed by Figure 2. *c* is defined as the number of the channels, *v* is the corpora's vocabulary size, *N* (*N* is the max length of the input sentence) is the length of input sentences, and *d* is the word embedding dimension. By looking up the pretrained multichannel word embeddings **D** ∈ *R* ^*c*×*v*×*d*^, the multichannel inputs **V** can be represented as a 3-dimensional array with size *c* × *N* × *d*; the subsequent convolutional layer would take **V** as input and extract the features.

### 2.3. Convolutional Layer

The convolution operation could be considered to apply different filters **W** ∈ *R* ^*c*×*h*×*d*^ to the *h*-word windows in each channel of the input **V**. Suppose **W** ^*i*^ ∈ *R* ^*h*×*d*^ donates the filter for channel *i* and **V** ^*i*^ ∈ *R* ^*N*×*d*^ is one of input word embeddings for channel *i*; a features **m**_*k*_ could be generated by (1), where **V** ^*i*^[*k* : *k* + *h* − 1] (the red and yellow parts in Figure 2) is generated by parallel connecting row *k* to row *k* + *h* − 1 in **V** ^*i*^, *f* is an activation function, *b* is a bias term, and ⊙ is element-wise multiplication

(1) $$\begin{array}{c} m_{k}=f\left(\;\overset{c}{\underset{i=1}{\sum }}V^{i}\left[\;k:k+h-1\;\right]⊙W^{i}+b\;\right). \end{array}$$

By applying an filter to each window in input sentence through (1), the model could produce a new feature **C** called feature map by

(2) $$\begin{array}{c} C=\left[\;m_{1},m_{2},m_{3},\ldots ,m_{N-h+1}\;\right]. \end{array}$$

Intuitively, convolutional layer is equal to applying filters on* n-grams* of input sentence. With different window size *h*, convolutional layer could extract various* n-grams* information.

### 2.4. Max-Pooling Layer

Max-pooling [26] operation by taking the maximum value over **C** (see (3)) brings two advantages: (1) it could extract the most important local features; (2) it reduces the computational complexity by reducing the feature dimension. A filter **W** would produce a feature **C** ^*∗*^ (see (1), (2), and (3)), and thus *M* filters would generate *M* features. All of these features are represented by **r** ^*∗*^ = [**C**_1_^*∗*^, **C**_2_^*∗*^, **C**_3_^*∗*^,…, **C**_*M*_^*∗*^]

(3) $$\begin{array}{c} C^{*}=\max\left(\;C\;\right). \end{array}$$

A single window size *h* can only capture fixed-size context information, by applying different window sizes, the model could learn more abundant features, suppose we use *K* to represent the number of window sizes, by concatenating the generated **r** ^*∗*^ for each window size, and the full feature **r** ∈ *R* ^*KM*×1^ (the second last layer in Figure 2) is represented by

(4) $$\begin{array}{c} r=\left[\;r_{1}^{*},r_{2}^{*},r_{3}^{*},\ldots ,r_{K}^{*}\;\right]. \end{array}$$

### 2.5. Softmax Layer for Classification

Before feeding distributed representation **r** to the last Softmax layer for classifying the DDI or PPI type, original features space is transformed into confidence space **I** ∈ *R* ^*O*×1^ by

(5) $$\begin{array}{c} I=W_{2}r, \end{array}$$

where **W**_2_ ∈ *R* ^*O*×*KM*^ can be considered as a transformation matrix and *O* is the number of classes.

Each value in **I** represents the confidence of the current sample belongs to each class. A Softmax layer can normalize the confidences to [0,1] and thus can view the confidence from the perspective of probability. Given **I** = [*i*_1_, *i*_2_,…, *i*_*O*_], the output of Softmax layer **S** = [*s*_1_, *s*_2_,…, *s*_*O*_]. The Softmax operation can be calculated by (6). Both *s*_*j*_ and *p*(*j*∣**X**) represent probability of an entity pair **x** which belongs to class *j*

(6) $$\begin{array}{c} s_{j}=p\left(\;j\;|\;x\;\right)=\frac{e^{i_{j}}}{\sum_{k=1}^{O}e^{i_{k}}}. \end{array}$$

### 2.6. Model Training

There are several parameters which need to be tuned during the training: the multichannel word embeddings **D**, the multifilters **W**, the transformation matrix **W**_2_, and the bias terms *b*. All the parameters are represented by *θ* = (**D**, **W**, **W**_2_, *b*). For training, we use Negative Log-Likelihood (NLL) in (7) as loss function (**y**_*i*_ is annotated label for the input sentence **x**_*i*_, and *L* is the minibatches size which means *L* samples will be fed to model in each training time). In order to minimize the loss function, we use gradient descent (GD) based method to learn the network parameters. In each training time, for *L* input samples 〈**x**_*i*_, **y**_*i*_〉, we firstly calculate the gradient (using the chain rules) of each parameter relative to loss and then update each parameter with learning rate *λ* by (8). It is notable that fixed learning rate *λ* would lead to unstable loss in training. In this work, we use an improved GD based algorithm Adadelta [27] to update the parameters in each training step; Adadelta can dynamically adjust the learning rate

(7) loss=∑i=1L−log⁡pyi ∣ xi,

(8) θ=θ−λ∂lossθ.

## 3. Experiments

In this section, we firstly demonstrate the preprocessing method for both train and test corpora in DDI and PPI tasks. Secondly, the experimental results on DDI and PPI tasks are reported, respectively, for each task, we start from a baseline model with one-channel randomly initialized word embedding, and then, we show the results of one-channel word embedding; after that, we conduct the experiments on multichannel CNN model. In discussion part, we analyze the effects of hyperparameters settings as well as the typical errors caused by MCCNN.

### 3.1. Preprocessing for Corpora

The standard preprocessing includes sentence splitting and word tokenise. If there are *n* entities in a sentence, then, *C*_*n*_^2^ entity pairs would be generated. To reduce the sparseness and ensure the generalization of features, we share the similar preprocessing method as [11, 14] by replacing two target entities with special symbols* “Entity1”* and* “Entity2*,*”* respectively, and entities which are not target entities in inputs are all represented as* “EntityOther*.*”* Table 2 demonstrates an example of preprocessing method.

The preprocessing method mentioned above may also produce some noise instances. For instance, entity pairs referred to the same name are unlikely to interact with each other. Such noise instances may (1) cause the imbalance distribution of the data, (2) hurt the performance of classifier, and (3) increase the training time. We define two rules to filter the noise instances. The rules are listed as follows.  Table 3 shows the examples of noise instance for the rules.

**Rule 1 .**

Entity pairs referred to the same name or an entity which is an abbreviation of the other entity should be removed.

**Rule 2 .**

Entity pairs which are in a coordinate structure should be discarded.

### 3.2. Evaluation on DDI Task

#### 3.2.1. Datasets

DDIExtraction 2013 challenge (https://www.cs.york.ac.uk/semeval-2013/task9/) provides the benchmark corpora and annotations for DDI task [28]. The main purpose of this task is to pursue the classification of each drug-drug interaction according to one of the following four types:* advice*,* effect*,* mechanism*, and* int*; therefore, DDI is a 5-label (four interaction types plus one negative type) classification task. We shortly describe each interaction type and give an example for each type:

1. *advice*: a recommendation or advice regarding the concomitant use of two drugs. For example, interaction may be expected, and* UROXATRAL* should not be used in combination with other* alpha-blockers*;
2. *effect*: a description for the effect of drug-drug interaction. For example,* Methionine* may protect against the ototoxic effects of* gentamicin*;
3. *mechanism*: pharmacodynamic or pharmacokinetic interactions between drug pairs. For example,* Grepafloxacin*, like other* quinolones*, may inhibit the metabolism of* caffeine* and* theobromine*;
4. *int*: an interaction simply stated or described in a sentence. For example, the interaction of* omeprazole* and* ketoconazole* has been established.
5. *negative*: no interaction between two entities. For example, concomitantly given thiazide* diuretics* did not interfere with the absorption of a tablet of* digoxin*.

The training and testing corpora in DDIExtraction 2013 consist of two parts: DrugBank and MedLine. A detailed description for these corpus could be found in Table 4. As can be seen from Table 4, our filtering rules are effective. In train datasets, the negative noise instances are reduced by 34.0% from 23665 to 15624 and only 22 out of 4020 (about 0.5%) positive instances are falsely filtered out. As for testing data, 35.0% of noise instances are discarded, while only 3 positive instances are mistaken. Such simple preprocessing method is beneficial to our system; especially it can reduce training time and avoid unbalanced classes.

#### 3.2.2. Pretrained Word Embedding

As mentioned before, five versions of pretrained word embeddings are used in MCCNN as shown in Table 5. There are 13767 words (some of drug entities consisted with multiwords are all considered as single words) in DDI corpus. As a result, unknown words in smaller PMC and MedLine can be* “made up”* by word embedding with larger vocabulary coverage such as Wikipedia and PubMed.

#### 3.2.3. Experimental Settings and Results

The experimental settings for DDI task are as follows: 200 filters are chosen for convolutional layer; minibatches size is set with 20; and window size *h* is set by 6, 7, 8, and 9, respectively. We select Relu as the activation function for convolutional layer due to its simplicity and good performance. Gaussian noise with mean 0.001 is added to the input multichannel word embedding, to overcome and prevent overfitting; we also add the weight constraint 5 to the last Softmax layer weight. Discussion section gives the details on parameter selection as well as the impact of the parameters.

Table 6 shows experimental results of baseline, one-channel, and the proposed MCCNN. As shown in Table 6, for each interaction type, we calculate the precision (*P*), recall (*R*), and the *f*-scores (*F*). We also report the overall micro-*f*-scores which has been used as a standard evaluation method in DDIExtraction 2013 challenge.

The baseline model utilizes randomly initialized word embedding, and the semantic similarity between words is not considered. Table 6 shows that one-channel with pretrained word embedding model performed much better than the baseline model and improved the overall *f*-scores from 60.12 to 66.90. This demonstrates that semantic information is crucial in DDI.

From Table 6, we can also find that, compared with one-channel model, MCCNN model achieved better results and improved the overall *f*-scores by 3.31%. For individual interaction type classification, MCCNN model also achieved the best *f*-scores. This demonstrates the effectiveness of the use of multichannel word embedding and richer semantic information.

We also trained the model on the corpus without preprocessing; the results could be found in Table 7. As we can see, preprocessing is important, which can improve the *f*-scores by 2.21% through reducing the potentially misleading examples.

Another aspect to note is that all three models behave worst on interaction type* “Int*,*”* such results are consistent with other systems [29–31], and the poor performance is mainly due to the lack of training samples (only 188 samples for training data and 96 samples for test data in Table 4).

In conclusion, (1) semantic information is important in DDI task, (2) rich semantic information can improve the performance, (3) preprocessing rules are crucial in DDI task, and (4) data scale would affect the model performance.

#### 3.2.4. Performance Comparison

In this section, we compare the proposed MCCNN model with the top 3 approaches in DDIExtraction 2013 challenge (FBK-irst [29], WBI [29], and UTurku [31]). We also compare with the recently [14] novel linear kernel based SVM method. All of the four systems use SVM as the basic classifier. Both the FBK-irst and Kim's system detected the DDI at first (binary classification) and then classified the interaction into a specific type (multilabel classification). Different from FBK-irst's one-against-all strategy, Kim et al. utilized the one-against-one strategy for DDI type classification. They claimed the strategy could reduce the effect of unbalanced classes. WBI and UTurku ignored strategies problem by using multiclass SVM. The characteristics of the four approaches and the result comparisons are all listed in Tables 8 and 9.

As we can see, feature engineering still accounts for a large proportion of these systems. The features like word-levels features, dependency graphs, and parser trees are commonly used. In addition, syntax and dependency analysis are not effective for long sentences. The proposed MCCNN is able to avoid these problems by using word embedding and CNN. As shown by Table 9, MCCNN performs better than other methods for detecting interaction types* “Advice*,*” “Effect*,*”* and* “Mechanism”* and further improves the state-of-the-art overall *f*-scores by 3.2%.

In addition, for interaction detection subtask (DEC), MCCNN achieved the second best *f*-scores compared to the FBK-irst's 80.0. DEC is a binary classification task, focusing on distinguishing the negative and positive instances. For most of the traditional methods, the most direct way is using cue words as they are not likely to be included in negative instances; in other words, “vocabulary gap” problem is not serious in these traditional methods. But in the problem of fine-grained interaction type classification, semantic information shows importance to classify different types. MCCNN showed its effectiveness on fine-grained classification by combing richer semantic information.

#### 3.2.5. Compared with Other CNN Based Models

It is notable that CNN was also utilized by Zhao et al. [32] recently; they combined traditional CNN and external features such as contexts, shortest path, and part-of-speech to classify the interaction type and achieved an overall *f*-scores 68.6 which was similar to our results. The differences between [32] and our model lie on two aspects: (1) feature engineering still plays an important part in [32] model, whereas our model demands no manually feature sets; (2) multichannel word embeddings in our model contain richer semantic information which has been proved to be much useful in fine-grained interaction classification task.

#### 3.2.6. Evaluation on Separated DrugBank and MedLine Corpus

Table 10 shows the performances of MCCNN on separated DrugBank and MedLine corpus. As shown in Table 10, MCCNN obtained *f*-scores 70.8 (compared to Kim's 69.8, FBK-irst's 67.6) on DrugBank and a sharp decline *f*-scores 28.0 (compared to Kim's 38.2, FBK-irst's 39.8). Reference [29] pointed out that such worse performance on MedLine might be caused by the presence of the cue words. From our point of view, the smaller number of training sentences in MedLine could also lead to the poor performances, as a proof, the MCCNN performed much better on MedLine (52.6) when trained on larger DrugBank and much worse (10.0) on DrugBank when trained on smaller MedLine in Table 10. As mentioned earlier, the scale of the data still has a great impact on the final results.

### 3.3. Evaluation on PPI Task

#### 3.3.1. Datasets and Pretrained Word Embedding

Two PPI datasets Aimed and BioInfer (http://mars.cs.utu.fi/PPICorpora/) are used to evaluate MCCNN. Aimed was manually tagged by Bunescu et al. [33] which included about 200 medical abstracts with around 1900 sentences and was considered as a standard dataset for PPI task. BioInfer [34] was developed by Turku BioNLP group (http://bionlp.utu.fi/clinicalcorpus.html) which contained about 1100 sentences. For corpora preprocessing, we do not use the filter rules in PPI task because of the limited size of corpus. The statistics of two datasets could be found in Table 11. We also report the vocabulary included in five pretrained word embeddings in Table 12.

#### 3.3.2. Changes of Performance from Baseline to MCCNN

For PPI experimental settings, the only difference from DDI task is the window size. Because the average sentence length in PPI task (42 in BioInfer, 36 in Aimed) is shorter than sentence length in DDI task (51), we set windows size *h* as 3, 4, 5, and 6.

Table 13 shows the experimental results of baseline, one-channel, and the proposed MCCNN on PPI task. We used 10-fold cross validation method for evaluation. As can be seen from Table 13, one-channel model performed much better than baseline model and improved the *f*-scores by 1.31% and 4.73% on Aimed and BioInfer, respectively. MCCNN achieved the best *f*-scores and improved the *f*-scores by 6.87% and 2.55% on Aimed and BioInfer when compared with one-channel.

#### 3.3.3. Performance Comparison

Table 14 shows the comparisons with other systems on Aimed and BioInfer corpus. Kernel methods have been proved efficient in recent researches. Reference [22] proposed a single convolutional parse tree kernel and gave an in-depth analysis about the tree pruning and tree kernel decay factors. Reference [11] made full use of the shortest dependency path and proposed the edit-distance kernel. It has been verified that a combination of multiple kernels could improve effectiveness of kernel based PPI extraction methods. References [23–25] proposed hybrid kernel by integrating various kernels, such as bag-of-word kernel, subset tree kernel, graph kernel, and POS path kernel; they all achieved competitive results on PPI task.

It is notable that the word embedding information was also integrated by Li et al. [2]. They assigned a category to each word by clustering the word embedding, which can be used as a distributed representation feature. They also made full use of brown cluster and instance representation by words clustering method. The relationship between two words is no longer a simple yes or no; words with similar meanings are clustered and assigned with the same class label. The methods are essential to weaken* “vocabulary gap”* and proved to significantly improve the performance in their experiments (7.1% and 4.9% *f*-scores improvement on Aimed and BioInfer compared with their baseline model). Through combining the other features such as bag-of-words and syntactic features, they obtained remarkable results on Aimed and BioInfer.

Distributed representation features proposed by Li et al. [2] could be considered as a* “hard”* assignment: a cluster label for each word, but the extracted features are still discrete. As a benefit from word embedding and CNN, the proposed MCCNN model is able to be trained in a continuous space and manual assignment is not necessary. Compared with existing kernel based methods, the baseline model yielded a comparable performance. By replacing the randomly initialized word embedding with pretrained one, the one-channel model achieved better results and improved the state-of-the-art *f*-scores by 3% on BioInfer corpora. Furthermore, by integrating multichannel word embedding, the proposed MCCNN model exceeded 2.7% and 5.6% compared with [2] approach on Aimed and BioInfer.

### 3.4. Discussions

In this section, we firstly investigate the effects of hyperparameters, and then we carefully analyze the errors caused by MCCNN as well as the possible solutions to errors.

#### 3.4.1. Hyperparameter Settings

The hyperparameters of neural network have great impact on the experimental results. In this work, three parameters including window size *h*, filter numbers *M*, and minibatches size need to be adjusted. To find the best hyperparameters, we split the training datasets into two parts: one for training and the other for validation. The basic method is to change one of the parameters while the other parameters remain unchanged. Filter numbers are set by [10,20,50,100,200,400], and the value range of minibatches size is [10,20,50,100]; in addition, windows size *h* is set by [3,5, 7,9, 11,13]. Experimental results show that the best settings for system are as follows: *M* is 200, minibatches size is 20, and *h* is 7 (7 in DDI task and 3 in PPI task). According to the suggestion that the best window size combination is usually close to each other by Zhang and Wallace [35], we set the windows size *h* as [5,6, 7,8] in DDI task and [3,4, 5,6] in PPI task.

Two methods are used to train a more robust model as well as prevent model from overfitting. The first method is to add Gaussian noise to the multichannel word embedding inputs. Considering the example in Table 2, the only differences of the three instances are the positions of Entity1, Entity2, and EntityOther; Gaussian noise could help to distinguish these instances. Experimental results showed that Gaussian noise can improve the performance by 0.5% in DDI task. In addition, according to [36], Gaussian noise could prevent overfitting. The other method is to add the weight constraint 5 to the last Softmax layer weight which could prevent overfitting.

#### 3.4.2. Errors Analysis

Subjected to the complexity and diversity of the biomedical expressions, extracting relations from biological articles remain a big challenge. In this subsection, we carefully analyze the errors caused by MCCNN and list the two typical errors as follows:

1. An input sentence is very long (more than 60 words), and Entity1 in this sentence is very close to Entity2.
2. An input sentence is very long (more than 70 words), and Entity1 in this sentence is far from Entity2.

As the only input for MCCNN is a whole sentence, Entity1 and Entity2 are likely to be included in the same word window if Entity1 is very close to Entity2. In addition, due to the long context, the irrelevant word windows also have the chance to be chosen, and noise windows could hurt the system's performance. In the second case, a fixed window size such as 7 might fail to capture long sentence context when two entities are far from each other. A possible solution to avoid the above two errors might introduce dependency parser or parse tree information that would be able to capture the syntax information no matter the distance of the two entities.

## 4. Conclusion

In this work, we focused on three issues in biological relation extraction. The first is the* “vocabulary gap”* problem that would affect the performance of the biological extraction system; the second is how integration of semantic information will improve the performance of the system; and the third is the investigation of a mean to avoid the manual feature selection. The first two issues could be solved by introducing word embedding, especially the multichannel word embedding. By integrating CNN with aforementioned multichannel word embedding, the third problem could be well solved, and the experimental results show that our proposed MCCNN is at least effective for the two typical types of biomedical relation extraction tasks: drug-drug interaction (DDI) extraction and protein-protein interaction (PPI) extraction. In error analysis section, we notice that the proposed MCCNN is not capable of dealing with long sentences. In our future work, we would like to design and evaluate our relation extraction system by making full use of multichannel word embeddings, CNN, and syntax information.

## 5. Implementation

We use Keras (https://keras.io/) to implement our model. The configurations of our machine are listed in Table 15. It takes about 400 seconds to finish an epoch in training and 21 seconds to predict the results during the test. In order to get the best result, 10 iterations over train corpus are usually required.

## Figures and Tables

**Table 1:** Statistics for five word embeddings (all with 200 dimensions).

	Vocabulary size	Training corpus
1	2515686	PMC
2	2351706	PubMed
3	4087446	PMC and PubMed
4	5443656	Wikipedia and PubMed
5	650187	MedLine

**Table 2:** An example for preprocessing of sentence *“Caution should be exercised when administering nabumetone with warfarin since interactions have been seen with other NSAIDs”* in DDI task. There are 3 entities in this example, and thus 3 entity pairs would be generated.

Entity1	Entity2	Generated inputs
Nabumetone	warfarin	Caution should be exercised when administering Entity1 with Entity2 since interactions have been seen with other EntityOther

Nabumetone	NSAIDs	Caution should be exercised when administering Entity1 with EntityOther since Interactions have been seen with other Entity2

Warfarin	NSAIDs	Caution should be exercised when administering EntityOther with Entity1 since interactions have been seen with other Entity2

**Table 3:** Examples of noise instance for defined rules; the mentioned entities are in italic.

Rule 1	*Anesthetics*, general: exaggeration of the hypotension induced by general *anesthetics*

Rule 2	To minimize CNS depression and possible potentiation, *barbiturates*, *antihistamines*, *narcotics*, *hypotensive* agents or phenothiazines should be used with caution

**Table 4:** Statistics for DDIExtraction 2013 challenge corpus. The entities pairs interacting with each other are labeled as positive, otherwise negative. The abstract indicates the number of article abstracts in datasets.

	Train			Test		
	DrugBank	MedLine	Overall	DrugBank	MedLine	Overall
Abstract	572	142	714	158	33	191
Positive	3788	232	4020	884	95	979
Negative	22118	1547	23665	4367	345	4712
Advice	818	8	826	214	7	221
Effect	1535	152	1687	298	62	360
Mechanism	1257	62	1319	278	24	302
Int	178	10	188	94	2	96

After preprocessing and filtering rules						
Positive	3767	231	3998	884	92	976
Negative	14445	1179	15624	2819	243	3062
Advice	815	7	822	214	7	221
Effect	1517	152	1669	298	62	360
Mechanism	1257	62	1319	278	21	299
Int	178	10	188	94	2	96

**Table 5:** Vocabulary included in five pretrained word embeddings.

	Vocabulary size	Word embedding
1	9984	PMC
2	10273	PubMed
3	10399	PMC and PubMed
4	10432	Wikipedia and PubMed
5	9639	Medline

**Table 6:** Experimental results of baseline, one-channel, and the proposed MCCNN on DDI task. *Baseline*: with one-channel randomly initialized word embedding. *One-channel*: with one-channel Wikipedia and PubMed word embedding.

	Baseline			One-channel			MCCNN		
	*P*	*R*	*F*	*P*	*R*	*F*	*P*	*R*	*F*
Advice	**89.39**	53.88	67.24	80.77	67.12	73.32	82.99	73.52	**77.97**
Effect	56.32	57.42	56.87	60.46	73.67	66.41	**67.03**	69.47	**68.23**
Mechanism	78.33	53.36	63.47	64.72	70.81	67.63	**85.00**	62.75	**72.20**
Int	**93.55**	30.21	45.67	82.05	33.33	47.41	75.51	38.54	**51.03**
Overall (micro)	70.00	52.68	60.12	66.50	67.31	66.90	**75.99**	65.25	**70.21**

**Table 7:** Performances of model with and without preprocessing.

	*F*-score
MCCNN (with preprocessing)	**70.21**
MCCNN (without preprocessing)	67.80

**Table 8:** Feature sets for four approaches.

Method	Feature sets
Kim	Word features, dependency graph features
	Word pair features, parse tree features
	Noun phrase constrained coordination features

FBK-irst	Linear features, path-enclosed tree kernels
	Shallow linguistic features

WBI	Features combination of other DDI methods

UTurku	Linear features, external resources
	Word features, graph features

**Table 9:** Comparisons with other systems on *f*-scores. ADV, EFF, MEC, and INT donate advice, effect, mechanism, and int, respectively, while DEC refers to interaction detection.

	ADV	EFF	MEC	INT	DEC	Overall
Kim	72.5	66.2	69.3	48.3	77.5	67.0
FBK-irst	69.2	62.8	67.9	**54.7**	**80.0**	65.1
WBI	63.2	61.0	61.8	51.0	75.9	60.9
UTurku	63.0	60.0	58.2	50.7	69.6	59.4

MCCNN	**78.0**	**68.2**	**72.2**	51.0	79.0	**70.2**

**Table 10:** Evaluation results (overall *f*-scores) on separated DrugBank and MedLine corpus. The first column corresponds to the training data set, while the first row corresponds to the test data set.

	DrugBank	MedLine
DrugBank	70.8	52.6
MedLine	10.0	28.0

**Table 11:** Statistics for Aimed and BioInfer datasets after preprocessing.

Datasets	Positive	Negative
BioInfer	2512	7010
Aimed	995	4812

**Table 12:** Vocabulary in pretrained word embedding.

	Aimed	BioInfer	Word embedding
All	6276	5461	—
1	5293	4666	PMC
2	5363	4712	PubMed
3	5404	4749	PMC and PubMed
4	5414	4762	Wikipedia and PubMed
5	4977	4328	MedLine

**Table 13:** Change of performances from baseline to MCCNN on Aimed and BioInfer datasets, respectively.

	Baseline			One-channel			MCCNN		
	*P*	*R*	*F*	*P*	*R*	*F*	*P*	*R*	*F*
Aimed	71.62	61.25	64.27	72.28	60.82	65.58	76.41	69.00	**72.45**
BioInfer	78.13	73.00	72.34	76.06	79.43	77.07	81.30	78.10	**79.62**

**Table 14:** Comparisons with other systems (*f*-scores) on Aimed and BioInfer.

	Aimed	BioInfer
Choi and Myaeng [22]	67.0	72.6
Yang et al. [23]	64.4	65.9
Li et al. [2]	69.7	74.0
Erkan et al. [11]	59.6	—
Miwa et al. [24]	60.8	68.1
Miwa et al. [25]	64.2	67.6

MCCNN (the proposed)	**72.4**	**79.6**

**Table 15:** Configurations of machine.

GPU	NVIDIA GeForce GTX TITAN X
CPU	Intel(R) Xeon CPU E5-2620 v3 @ 2.4 GHz
System	Windows 7
memory	8 G
